# Preliminary Assessment of the Acute Effects of Far Infrared-Emitting Garments: What Are the Possible Implications for Recovery and Performance?

**DOI:** 10.3390/life13101998

**Published:** 2023-09-30

**Authors:** Alexander Bertuccioli, Roberto Cannataro, Marco Gervasi, Piero Benelli, Aurora Gregoretti, Mirko Ragazzini, Marco Neri, Chiara Maria Palazzi, Marco Cardinali, Giordano Zonzini

**Affiliations:** 1 Department of Biomolecular Sciences, University of Urbino Carlo Bo, 61029 Urbino, Italy; 2 Microbiota International Clinical Society, 10123 Torino, Italy; 3 Galascreen Laboratories, University of Calabria, 87036 Rende, Italy; 4 Research Division, Dynamical Business and Science Society, DBSS International SAS, Bogota 110311, Colombia; 5 AIFeM (Italian Medicine and Fitness Federation), 48121 Ravenna, Italy; 6 Department of Internal Medicine, Infermi Hospital, AUSL Romagna, 47921 Rimini, Italy

**Keywords:** FIR, FIR garments, hemodynamics, thermoregulation, performance, recovery, super-compensation, BIVA

## Abstract

Technical clothing has recently been brought into the spotlight as one of the most promising tools to improve sports performance and to enhance sports recovery. Among technical clothing items, garments engineered to emit far infrared (FIR) spectrum frequencies have come to the fore as a treatment for pain, muscle fatigue, and tissue healing due to their potential antioxidative and anti-inflammatory properties, with applications not only during recovery phases but also in the active phases of exercise. These garments, composed of fibers mixed with noble metals and/or bioceramics that respond to body infrared frequencies by returning an FIR emission backwards, are thought to improve muscle oxygenation and therefore recovery. In this double-blind, randomized, placebo-controlled, crossover study, ten male trail running athletes wore a whole-body-covering suit marketed as Accapì-FIR (Bruno Chiaruttini S.r.l., Rezzato, BS, Italy), while a total body suit with the same polyester fiber without metal components was used as control for the intervention. Parameters such as weight, height, bioimpedance parameters (BIVA), lactate from capillary sampling, salivary cortisol, and average temperatures of different body areas were obtained before and after a twelve-minute incremental work run protocol on a treadmill whilst wearing the two kinds of garment. Using the intervention suit, compared to control, statistically significant reductions in BIVA parameters such as body resistance (−6.7%) and reactance (−5.4%) were observed before and after exercise while a greater, but not significant, weight reduction was observed with the intervention suit. Decrease in resistance could be the result of a different distribution of fluids and ions due to FIR influence on capillary and superficial circulation, leading ultimately to more efficient management of body heat and promoting recovery and supercompensation. Further studies on larger samples will be necessary to confirm and clarify these results.

## 1. Introduction

The search for techniques and solutions that can optimize performance and recovery processes is an element of great interest in sports medicine. Among the many feasible solutions, technical clothing has enjoyed one of the greatest successes in recent years, producing technological solutions, for example, capable of modulating body temperature and humidity, promoting better blood circulation and muscle contraction, and improving performance and recovery. Among the various technologies developed, that which makes it possible to exploit garments specially engineered to emit frequencies falling within the far infrared (FIR) spectrum represents a potential yet to be explored. The acronym FIR refers to a specific band of electromagnetic radiation, defined differently in different classification models. One of the most widely used classifications is the one proposed by Byrnes et al., which places the FIR between 15 and 1000 μm of the infrared spectrum (IR), the mid-wavelength infrared (MWIR/IR-C DIN) between 3 and 8 μm and the long-wavelength infrared (LWIR/IR-C DIN) between 8 and 15 μm [1]. In disagreement with this classification, those widely used in physical and rehabilitation medicine place the FIR between 3 and 1000 μm according to the classification proposed by Zati and Valent [2]. Every living subject (including the human organism) or inanimate object with a temperature higher than absolute zero generate an environmental emission of FIR radiation not perceptible with the human visual system but rather detectable in the form of thermoception by the integumentary system. The FIR radiation is therefore perceived as radiant heat, penetrating a depth of up to 4 cm into tissue [3], a level at which, according to some authors, it could theoretically exert biological effects at the cellular and molecular level [3]. Various technological solutions based on the emission of FIR are used in physical medicine and rehabilitation for the treatment of various pathological and para-physiological conditions characterized by elements such as pain, muscle fatigue, joint fatigue, wound healing, heart failure, sleep disorders, lactation, dysmenorrhea, and others [3]. Numerous studies that have investigated the biological mechanisms involved in the use of FIR technology have been carried out in vitro and in animal models, highlighting potential applications in the management of inflammation [4], oxidative stress [5], muscle–tendon efficiency, and the integumentary system [6], and in the modulation of microbial development [7]. Based on these data, various proposed applications have been developed to delay muscle fatigue [8] and promote blood circulation with particular reference to the peripheral microcirculation (within the tissues reachable by the penetration depth of the FIR frequencies) [9,10] and cerebral microcirculation [11]. According to some authors, these effects could also act on the metabolic profile of the regions reached [9]. Numerous applications of FIR technology exploit means other than technical clothing [12], and FIR cabins, lamps, and other types of emitters of different shapes and crafts have been created [3]. In a profoundly dynamic scenario such as sports, applications made with fabrics are generally the most versatile and usable, not only in the recovery phases but also in active phases on the field. The fabrics used in the production of technical products are generally composed by incorporating different technological fibers or bioceramic powders (such as noble metals, magnesium, silica, etc.) directly into the fibers used in the manufacture of the fabric, or they can be intertwined with the fibers (in different proportions), or they can be applied in the form of “plates” on specific areas of the fabric. Once irradiated with the infrared frequencies emitted by the body, the noble metals and/or bioceramics respond by returning a FIR emission [13]. In the absence of additional heating effects to the ambient temperature [14], the thermal energy emitted by the body in the form of IR is absorbed and re-emitted through the mechanisms of convection and conduction towards the body in the wavelength of the FIR emission. Based on this mechanism, various authors suggest a use not merely aimed at temperature control but rather aimed at obtaining a biological response, describing effects such as the improvement in muscle oxygenation [15,16], probably due to the increase in peripheral blood flow [15], with detectable effects on resting metabolic rate [17] and subjective parameters of sleep [18]. Additionally, the review carried out by Bontemps et al. has recently analyzed the current knowledge available on FIR technology exhaustively [19], defining the main known mechanisms of action, based on findings in the medical and physiotherapy fields, specifically the modification of the thermoregulatory and hemodynamics. At the same time, there are still several questions regarding applications in sports, where the optimization of physical performance and post-exercise recovery are the areas of greatest interest [19]. This preliminary work was carried out to analyze whether the use of technical clothing made with FIR fabrics against a control fabric during a short and intense physical activity could imply effects on bioelectrical parameters of the human organism such as resistance (Rz) and reactance (Rx), and of the humoral parameters generally found in the field.

## 2. Materials and Methods

This preliminary study was performed in a double-blind, randomized, placebo-controlled, crossover study evaluating trail running athletes subjected to high-intensity activity under controlled conditions. During the training, the athletes wore technical clothing made with FIR fabrics or technical clothing made with conventional materials. The study was conducted following the rules of good clinical practice established by the Declaration of Helsinki and following the European Union Directive 2001/20/EC, according to the protocol approved by the Ethics Committee for Human Experimentation of Urbino University Carlo Bo (no. of approval 60_15marzo2023).

### 2.1. Participants

The enrolled subjects were ten male recreational trail running athletes with a mean age of 40.9 (±5.9) years, mean weight of 78.3 (±8.9) kg, mean height of 1.76 (±0.05) cm, and mean BMI of 25.3 (±2.9) kg/m^2^, as indicated in Table 1. Eating habits were investigated, verifying that the selected subjects had a uniform food profile suitable for the Mediterranean diet [20]. We chose subjects with a performance level related to age that was average based on the data present in the literature [21]. Furthermore, within this age group we assumed that the participants represented the majority of the athletic population, so in fact we excluded those of the greatest height, younger people under 20 years old, and older people above 60 years old, who represent the minority of athletes. The selected athletes had an average running experience of 20 +/− 3.8 years, in the last competitive season no participants rested for more than a month and they regularly raced competitively.

### 2.2. Inclusion and Exclusion Criteria

Only subjects with a valid sports medical certification for competitive activity, competitive practice of trail running for at least five years, and an average level of training to guarantee participation in competitive events over distances greater than 20 km with differences in height greater than 500 m were included. Exclusion criteria considered were for the use of medicines, drug use, alcohol abuse, inadequate possibility of physical/muscular recovery following competitions and training, injuries suffered in the last 60 days not fully recovered (with medical documentation), unfitness to practice competitive physical activity (even temporary), finding of paramorphism, pains of any kind.

### 2.3. Evaluated Products

**Figure 1:**
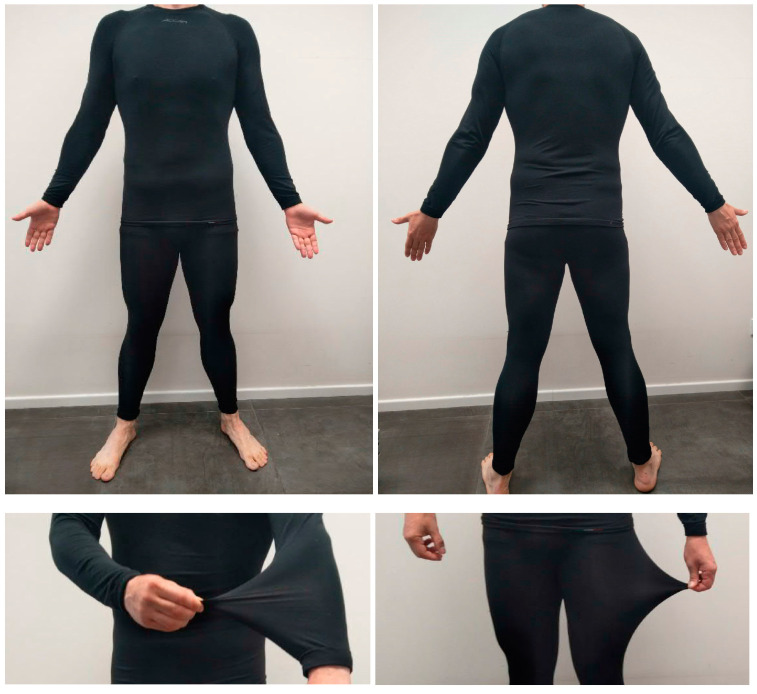
Fit and extensibility of the bodysuit.

The product tested during the evaluation is a whole-body covering suit marketed as Accapì-FIR (Bruno Chiaruttini S.r.l., Rezzato, BS, Italy). The product is made of polyester fibers that contain noble metal powders which, when excited by the infrared frequency produced by the body, emit a constant infrared emission between 5 and 20 µm, producing emissions in the MWIR, LWIR, and FIR frequencies. A total body suit made by the same manufacturer with the same polyester fiber without metal components was used as a control product, obtaining indistinguishable garments. Both products were manufactured in compliance with the ISO 10993-1 standard for cytotoxicity (UNI EN ISO 10993-5:2009, Report 4983-20 of 16 July 2020), skin irritation (UNI EN ISO 10993-10:2013, Report 8742-20 of 22 December 2020), and allergic potential from contact dermatitis (rLLNA) (UNI EN ISO 10993-1:2013, Report 8741-20 of 22 December 2020), due to the absence of cytotoxicity, sensitization reactions, and irritation. For subjects with a BMI between 22 and 25, we used the L size and between 25.1 and 28 we used the XXL size to guarantee homogeneous wearing conditions based on the evaluations made when the subjects tried the fabrics. The technical characteristics of the product are summarized in Table 2, and the fit of the bodysuit is represented in Figure 1.

### 2.4. Evaluation Scheme

The enrolled subjects received an anonymous kit containing the Accapì FIR technical clothing and the control technical clothing, marked with an alphanumeric code so that the two could not be distinguished by the subjects being evaluated or the examiners. The subjects were acclimatized in a controlled laboratory environment (temperature 22°, humidity 50%) for 30 min [22] wearing the FIR and control products, respectively. Once acclimatized, subjects were made to undress long enough to detect their weight, height, bioimpedance parameters (BIVA), lactate from capillary sampling, salivary cortisol, and average temperatures of different body areas (specifically, the shoulder, hip, knee, ankle, and medial thigh) using thermography, reflecting the work of Amighetti et al. [22]. We chose to monitor the joints and the muscle lodge most involved in this type of athlete and therefore potential sites of inflammatory processes. Weight and height were measured with a mechanical column scale SECA 700 Eye Level Beam (SECA North America, Medical Measuring Systems and Scales, 13601 Benson Avenue, Chino, CA 91710, USA), using an approximation of 50 g for the weight and 0.1 cm for the height according to the standard methodologies defined in the literature [23]. Utilizing BIVA analysis, the impedance components resistance (Rz) and reactance (Xc) were detected with a bioimpedance analyzer (BIA 101 BIVA PRO, Akern, Florence, Italy) [24,25,26,27,28,29,30], processing the data obtained with the Bodygram Dashboard^®^ software (Akern, Florence, Italy) [27,28,29,30], using BIATRODES electrodes (Akern, Florence, Italy) [27,28,29,30]. Before performing the BIVA, the subjects were placed supine on a non-conductive surface for at least 5 min, allowing for a homogeneous distribution of fluids. The lower limbs were abducted with an opening at 45° with respect to the midline of the body, and the upper limbs were abducted at 30° with respect to the trunk, verifying the absence of contact between the lower limbs, upper limbs, and trunk, and with any potentially conductive object. The skin was cleansed with alcohol at the points of application of the four electrodes (two injectors and two detectors), which were positioned on the back of the right hand, in correspondence with the radioulnar joint and the metacarpophalangeal joint of the third finger for the upper district, and on the back of the foot, one on the tibiotarsal joint and the other in correspondence with the metatarsophalangeal joint of the third for the lower section [24,25,26,27,28,29,30], respecting the distance of 5 cm from each other. The data obtained were processed using the Bodygram Dashboard software by analyzing the direct values and obtaining relatively derived estimates. Lactate was detected by performing a capillary puncture with an Accu-Check safe-T Pro Uno (Roche, Basel, Swiss) disposable sterile device [31,32] using Lacto Spark test strips (Sensacore, Hyderabad, India) on Lacto Spark analyzer (Sensacore, Hyderabad, India) [33]. The thermographies were carried out by preparing a workstation that allowed standardization of the subject’s positioning through visual aids; the images were acquired with a FLIR Lepton 3.5 sensor (Teledyne FLIR LLC, Wilsonville, OR, USA) [34]. The salivary sampling for cortisol detection was carried out with a specific kit (Microtrace, Novafeltria, Italy) [35] according to the instructions provided by the manufacturer; the freshly collected samples were placed in the freezer to await analysis. The instruments used were calibrated before each laboratory session according to the instructions provided by the manufacturer. After carrying out the initial measurements, the subjects donned the technical garments and waited at rest for a further 30 min of acclimatization. Once this phase was completed, the incremental workout protocol on Treadmil Run Excite Jog 500 (Technogym s.p.a, Cesena, FC, Italy) [36] began. The workout started at 8 km/h for 2 min, then increased the speed by 2 km/h every 2 min until reaching 18 km/h for a total time of 12 min. Once the workout protocol was completed, the technical clothing was removed, the lactate and thermographs were detected after 15 min to acclimatize and return to a state of calm, and all other parameters were measured again; specifically, weight, Rz, Xc, thermographs, lactate, and salivary cortisol. The duration of the evaluation protocol was an average of 100 min for each subject. After a 7-day recovery period, in which subjects abstained from physical activity, the protocol was repeated with the garment not used in the first evaluation. The statistical analysis of the results was carried out, and the measured variables reported as mean and standard deviation. The variations in the variables (∆) were expressed as the difference between the mean at T0 and T1. To verify if the use of the Accapì tissue was significantly associated with a different variation in weight, Rz, Xc, lactate, salivary cortisol, and temperatures, multiple analyses of variance (MANOVA paired data) were performed using time (paired measure) and group (binary between factor) as predictors. The significance level was 0.05 and the analyses were performed using Excel 365 (Microsoft, Redmont, WA, USA) [28] and SPSS 22.0 (IBM, Armonk, NY, USA) [28].

## 3. Results

In Table 3, the variables measured in pre- and post-exercise conditions are reported as mean ± standard deviation for both the control and treated groups. The MANOVA analysis shows a significant effect of the time factor (F20;19 = 19.28; *p* < 0.001); the changes over time are significantly different, considering the set of dependent variables (F20;19 = 3.11; *p* = 0.048). The subsequent univariate analyses are reported in Table 3.

**Figure 2:**
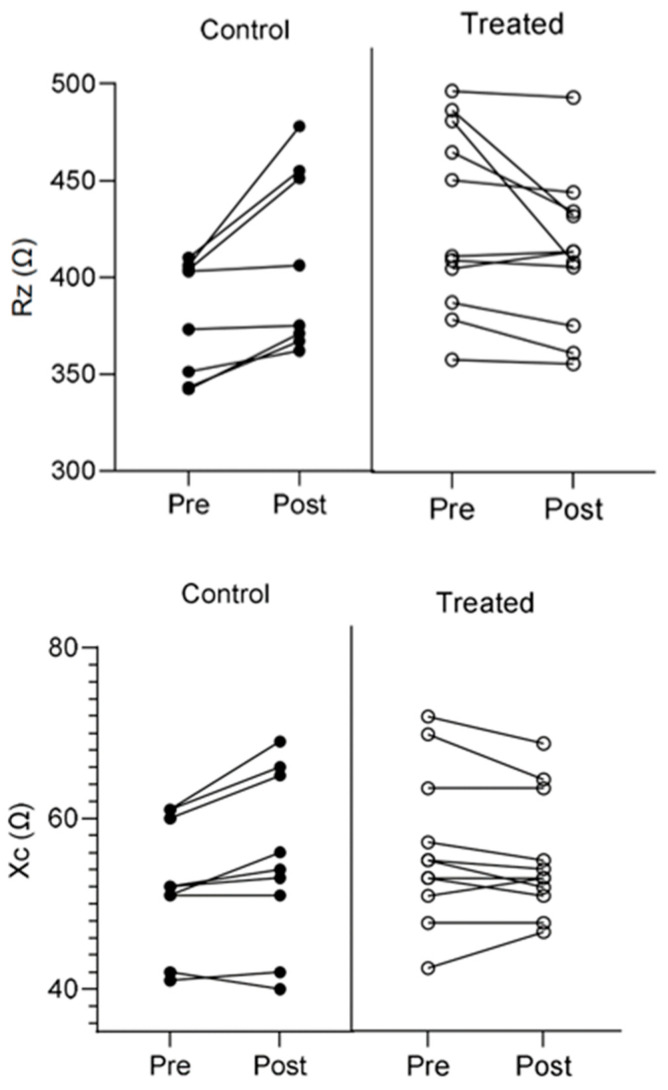
Rz and Xc detected with the Accapì FIR fabric and the control fabric.

The analysis of anthropometric parameters revealed a weight variation significantly different from zero (*p* < 0.001) but overlapping (*p* = 0.138) between the two evaluation sessions (−0.3% vs. −0.5%) when using the Accapì FIR suit and the control product, respectively, with a slightly higher weight reduction using FIR fabrics. The proposed exercise increased lactate (*p* = 0.034) and cortisol (*p* < 0.001) levels for both groups, which was non-significant between the two groups (*p* = 0.186 and *p* = 0.639, respectively). The analysis of bioimpedance parameters suggests a more complex situation. Using the Accapì FIR suit yielded statistically significant reductions in Rz (−6.7%) and Xc (−5.4%) between pre- and post-exercise. In contrast, for the bioimpedance values in the control group, an increase is observed in Rz (+4%) and Xc (+2.5%). The surface temperature of the various body districts shows a peculiar behavior; when wearing the FIR fabrics, there was a minor increase and consequently a minor reduction in the temperature upon suspension of the activity compared to the control fabrics, where instead, the situation is opposite. The most relevant parameters are summarized in Figure 2.

## 4. Discussion

**Figure 3:**
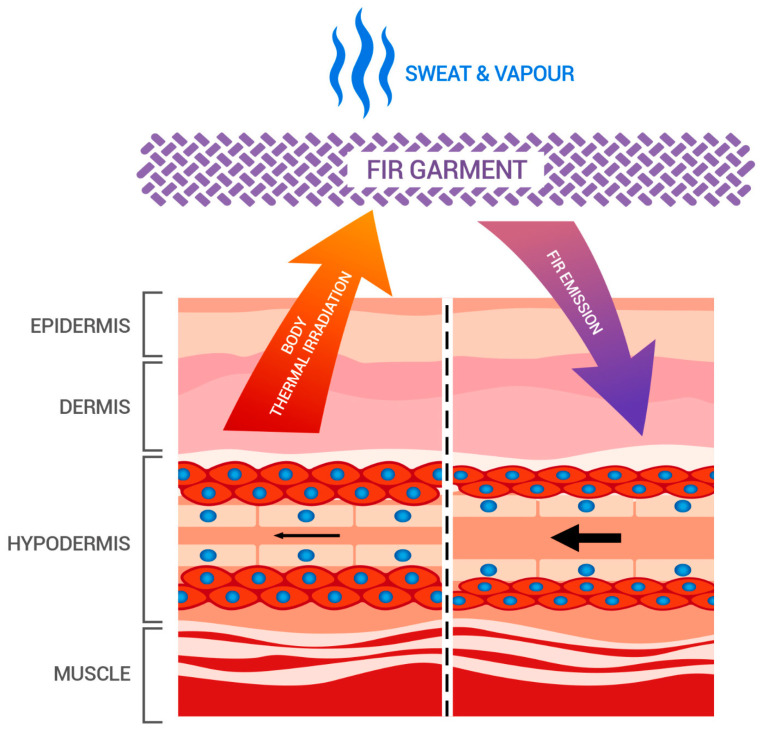
Combination of hemodynamic and thermoregulatory effect.

In this preliminary crossover study, carried out in a double-blind, randomized and placebo-controlled fashion, we examined the acute effects of wearing a suit made of fabric with FIR technology (Accapì FIR) 30 min before and during the performance of intense physical exertion, compared to an indistinguishable control fabric with the same proportion of fabric composition (73% polyester—22% polyamide—5% elastane) but in the presence of noble metals and bioceramics. The results that emerged suggest that the use of the material with FIR technology is associated with a significant variation (time × group *p* < 0.001) in body resistance (Rz) compared to the control fabric. In particular, we found a reduction in Rz in the treated group while there was an increase in the control group. In such a situation, following fluid loss and acute response to exercise, one would expect an increase in Rx and essentially unchanged Xc as it occurs substantially using control tissue. It should also be noted that the reduction in resistance observed in the Accapì FIR group was accompanied by an acute weight loss which was small but greater than that of the control group, which instead showed a small increase in resistance consistent with the performance of physical activity and relative weight loss through sweating. The slight increase in reactance in the control group is consistent with what has been shown in patients undergoing hemodialysis, where the variations in bioimpedance indices were inversely related to the volume of body fluid removed [37]. In contrast, as far as the treated group is concerned, the decrease found poses interesting questions that deserve to be investigated in the future to clarify whether this is an artifact or—considering the opposite behavior of the Rz compared to the control group—a phenomenon related to hemodynamic variations or the biological effects of FIR emissions. This seems to indicate an effect of these emissions on the dynamics of body fluid distribution, possibly due to hemodynamic and vasoactive effects, which have also been described in the literature following the application of FIR technologies [10,15,19]. This effect could be explained based on the ability of FIR tissues to act in the blood circulation [10,15] via the penetrating ability of FIR frequencies [2,19], as described above. Based on the data presented and the theoretical rationale proposed by various authors, it should correlate with implementation in the dynamics of peripheral circulation (e.g., by intervening in the capillary and superficial circulation), which can also be associated with the long-term, more efficient, management of body heat. A representative combination of hemodynamic and thermoregulatory effects is summarized in Figure 3. However, due to the small number of participants, we were not able to observe significant differences in body weight between the two groups, and we did not perform a real-time analysis of sweating levels, limiting our assumptions.

On the other hand, the evaluation of the temperatures in the different districts of the skin surface seems to underpin that by wearing the fabric with FIR technology, lower starting skin temperatures and lower reductions after physical stress with better thermal stability are observed on average, in contrast to the group control where there are higher starting temperatures and more significant reductions following physical stress with a greater exertion. An even more relevant factor, if one considers zones of considerable importance, in particular, the temperatures of the shoulder and quadriceps, is the regional mapping of perspiration rates following physical activity [38]. If these findings are confirmed in studies with a larger sample size, the potential role of the Accapì FIR suit in promoting better thermal stability and, consequently, better thermoregulation, a critical factor in the practice of intense physical activity, could be explored [39]. The results of this preliminary assessment of the suit containing noble metals with FIR emission capability deserve further investigation, as their use may be beneficial in certain environmental conditions, and particularly during physical activity [5,16,19,40].

In the present study, the results are inconclusive as to whether the changes found could benefit performance. However, if future studies confirm our hypotheses on hemodynamic and thermoregulatory effects, these garments could also prove useful in recovery and supercompensation processes. Furthermore, given that bacteriostatic and cooling effects (compatible with thermoregulatory properties) have been documented for FIR fabrics, their use could significantly improve the comfort of athletes during prolonged exercise [7,19].

## 5. Conclusions

In this preliminary evaluation, we considered the variation in bio-impedance parameters, lactate and cortisol values, and surface temperatures of certain skin areas that occurs when using a fabric (Accapì FIR) capable of producing, when stimulated by body heat, an emission of FIR infrared according to the classification proposed by Zati and Valent [2]. The results show that the use of the Accapì FIR suit is associated with a reduction in body resistance (Rz) and reactance (Xc) compared to the control fabric. In addition, when wearing the suit with FIR technology, we observed lower baseline skin temperatures and smaller post-exercise reductions compared to the control, where baseline temperatures are higher and post-exercise reductions are more significant. These effects can generally be attributed to the different distribution of fluids and ions that allows the body to offer less resistance to the passage of the microcurrent used in bioimpedance measurements. Further studies with larger samples and greater detail will be necessary to clarify the efficacy associated with the use of FIR fabrics, investigating all aspects of this technology, and defining potential applications.

## Figures and Tables

**Table 1:** Characteristics of enrolled participants.

Characteristics of the Participants	
Age	40.9 (±5.9) years
Weight	78.3 (±8.9) kg
Height	1.76 (±0.05) m
BMI	25.3 (±2.9) kg/m^2^
Chest circumference	99.75 (±6.4) cm
Waist circumference	87.75 (±8.2) cm
Hip circumference	100.25 (±3.9) cm

**Table 2:** Technical characteristics of the tested products.

Parameter	Value
FIR fabric composition	73% polyester—22% polyamide—5% elastane
Metallic and bioceramic component of the FIR fabric	<1 g/m^2^
Control fabric composition	73% polyester—22% polyamide—5% elastane
Metallic and bioceramic component of the control fabric	0 g/m^2^
Characteristics common to the two fabrics	
Compression	the fabric does not exert compression
Fabric thickness	0.692 mm
Density	133.7 gr/mq
Fabric extensibility	90%
Porosity	0.022 mmq
Resistance to conductive heat transfer of fabrics (RCT)	0.95 KW/mq
Air permeability (RET)	9
Creation of fabric structure	Seamless Technology
Bodysuit weight size L	223 g
Bodysuit weight size XXL	265 g

Density: number of warp or weft threads per unit length, referred to as warp density and weft density; porosity: measurement of the spaces between one mesh and another; RCT: resistance to conductive heat transfer of fabrics indicates the degree of thermal insulation of a garment (the higher the RCT, the more the garment will retain body heat, thermally insulating it, while garments with a low RCT will tend to release heat more easily); RET: the quantity in volume of air (or vapor) that passes through a square meter of fabric in one second under a given pressure difference, a parameter influenced by spacing between the warp and weft threads, the structure of the weave, and the porosity of the row.

**Table 3:** Values of the variables (mean ± standard deviation) measured in pre- and post-exercise conditions.

	Treated			Control				
	T0	T1	Var (%)	T0	T1	Var (%)	*p* (Time)	*p* (Time per Group)
Weight	78.3 ± 8.9	77.9 ± 9.1	−0.5%	78.2 ± 9.3	78 ± 9.3	−0.3%	<0.001	0.138
Rz	393.4 ± 61.9	366.9 ± 46.0	−6.7%	403.0 ± 36.3	419.0 ± 43.9	4.0%	0.335	<0.001
Xc	54.2 ± 10	51.3 ± 7.8	−5.4%	55.1 ± 6.5	56.5 ± 7.7	2.5%	0.205	0.001
Lactate	5.1 ± 3.9	5.7 ± 1.2	11.5%	4.7 ± 2	7.1 ± 1.1	50.3%	0.034	0.186
cortisol	2.1 ± 1.9	3.1 ± 2.4	46.3%	2.3 ± 2.2	3.1 ± 2.3	35.1%	<0.001	0.639
T shoulder	33.1 ± 3	32.5 ± 2.8	−1.8%	35.3 ± 1.1	33.1 ± 1.3	−6.0%	0.044	0.24
T hip	31.7 ± 2	31.9 ± 1.7	0.5%	33.2 ± 1.6	31.5 ± 1.2	−5.1%	0.158	0.093
T knee	31.6 ± 1.9	31 ± 2.6	−2.0%	32.7 ± 1.7	31.1 ± 1.3	−4.9%	0.102	0.463
T ankle	30.2 ± 1.4	30.5 ± 1.9	1.1%	30.9 ± 1.5	29.1 ± 0.9	−5.8%	0.062	0.009
T quadriceps	30.1 ± 1.2	30.1 ± 1.3	−0.3%	31.5 ± 1.5	30 ± 1.2	−4.8%	0.009	0.018

## Data Availability

Data related to this manuscript can be made available from the corresponding author upon reasonable request.
